# Evaluating the efficacy of tryptophan fluorescence and absorbance as a selection tool for identifying protein crystals

**DOI:** 10.1107/S1744309110002022

**Published:** 2010-02-27

**Authors:** Harindarpal S. Gill

**Affiliations:** a Case Western Reserve University, Department of Physiology and Biophysics, The Protein Expression Purification Crystallization Core, 10900 Euclid Avenue, Robbins Building E549, Cleveland, OH 44106-4970, USA

**Keywords:** tryptophan fluorescence, tryptophan absorbance, protein crystal identification

## Abstract

The environment of individual tryptophans in known protein structures and the effectiveness of four commercial robotic UV microscopes to illuminate tryptophan-containing protein crystals by either tryptophan fluorescence (epi-illumination) or absorbance (transmission) are evaluated. In agreement with other studies, tryptophan residues are found on average to be largely buried in protein structures (with ∼84% of their surface area buried) and to be surrounded by partially polar microenvironments (with ∼43% of their surface area covered by polar residues), which suggests an inherent degree of fluorescence signal quenching. In bacterial genomes, up to one-third (∼18.5% on average) of open reading frames are deficient in tryptophan. In the laboratory, because of the attenuation of UV light by the media commonly used in sitting-drop and hanging-drop crystallization trials, it was often necessary to simplify the light path by manually removing or inverting the supporting media. Prolonged exposure (minutes) to UV light precipitates some protein samples. The absorbance spectra of many commercially available media in crystallization trials are presented. The advantages of using tryptophan absorbance over fluorescence for characterizing crystals are discussed.

## Abstract 2

The effectiveness of using ultraviolet microscopes to illuminate protein crystals in high throughput screens is evaluated.

## 1. Introduction

Protein crystallization is an empirical science and requires that for each protein, crystallographers test thousands of solution compositions to find one that is optimal for generating crystals with well formed lattices that diffract X-rays. As structural genomics consortia and other laboratories aim to solve the three-dimensional structures of proteins on a genome-wide scale, a number of robotics have been marketed to screen and optimize a vast array of solution conditions in order to crystallize the proteins, which is a prerequisite for structure determination. These robots significantly minimize the amount of protein required for screening (referred to as low-volume protein drop setting), visually monitor crystal growth or identify provisionally optimal conditions and rapidly formulate new solutions to further optimize crystallization (D’Arcy *et al.*, 2004 ▶; McPherson, 2004 ▶; Newman *et al.*, 2008 ▶). But how do the robots automatically and systematically identify the growth of a protein crystal *versus* a salt crystal or non­proteinaceous object in a trial drop? The answer is that full automation has yet to be achieved; it is primarily blocked by a lack of methods to identify preliminary crystallization hits quickly. Current robots that image each trial drop at best implement Fourier-based software programs that identify the edges or analyze the textures of the objects in a drop (Spraggon *et al.*, 2002 ▶; Wilson, 2002 ▶; Cumbaa *et al.*, 2003 ▶; Berry *et al.*, 2006 ▶; Walker *et al.*, 2007 ▶; Watts *et al.*, 2008 ▶). However, these technologies cannot by themselves differen­tiate between salt crystals, amorphous material and protein crystals.

Traditionally, protein crystallographers make educated guesses as to the nature of a crystal by looking for dull edges on a well formed crystal and comparing its putative point-group symmetry, which suggests its space group, with their individual experience of working with other protein crystals. This approach, even for the experienced, is far from systematic and accurate. Crystallographers also sometimes miss microcrystal showers that are either mistaken for precipitated protein or have been buried by actual precipitate. Birefringence has been a useful and aesthetically pleasing property of crystals, helping to illuminate microcrystals in a drop (Echalier *et al.*, 2004 ▶). However, birefringence mostly helps to check for the uniform lattice of a non­composite crystal; it does not distinguish between salt crystals and protein crystals. Crystallographers then continue to manually and invasively dismantle the hanging-drop or sitting-drop crystallization experiment; they remove the cover slip that supports the trial drop in order to stain the crystal with dye, such as methyl violet, or to test the crystal for fragility by crushing it, both of which are signatures of protein. Even here the conclusion can be ambiguous and they have destroyed that crystal. Moreover, the typical drop in crystallization trials has become very small in order to minimize the amount of protein needed for screening. Each drop is now between 25 and 300 nl in high-throughput screening experiments, making handling and visual inspection more difficult. Lastly, crystallographers finally patiently mount each remaining crystal (if large enough) onto the X-­ray generator and observe the resulting diffraction pattern, which effectively characterizes the nature of the crystal. Because we lack a quick and systematic approach, the field of automation has yet to implement an effective scoring scheme to evaluate hits in crystallization trials.

An emerging technology in the field of protein crystallography is the development of the ultraviolet (UV) microscope. UV microscopes take advantage of the fluorescence of tryptophan residues under UV light to illuminate protein crystals. Tryptophans, and to a much lesser extent tyrosines and phenylalanines, fluoresce around 353 nm when excited with UV light at 280 nm. One of the first commercial UV-light microscopes to distinguish protein crystals from salt crystals was implemented by the company Korima (referred to here as microscope 1; Judge *et al.*, 2005 ▶). However, the principles of UV fluorescence had been implemented previously to center protein crystals in the X-ray beam (Pohl *et al.*, 2004 ▶). In more recent years, fluorescent dyes have also been used in cocrystallization experiments to help to identify protein crystals (Kettenberger & Cramer, 2006 ▶; Forsythe *et al.*, 2006 ▶; Groves *et al.*, 2007 ▶).

Studies have shown that UV light can be damaging to proteins by photolysis or photo-oxidation mechanisms (Dose, 1968 ▶; Permyakov, 1993 ▶; Vernede *et al.*, 2006 ▶; Kehoe *et al.*, 2008 ▶). Studies have also shown that fluorescence can be quenched by various mechanisms. For example, in the case of the nonradiative Förster resonance energy transfer (FRET) mechanism, a chemical group, usually at a distance of 15–60 Å from the tryptophan, accepts the energy that would otherwise be emitted at ∼353 nm (Permyakov, 1993 ▶; Lakowicz, 2006 ▶). Other examples include collisional mechanisms (*i.e.* quenching by diffusion of, for example, molecular oxygen from the solvent; Lakowicz, 2006 ▶), electron-transfer mechanisms [*i.e.* quenching by disulfide bridges, glutamine (Chen & Barkley, 1998 ▶), asparagine, glutamate, aspartate, cysteine and histidine residues (Chen & Barkley, 1998 ▶; Permyakov, 1993 ▶) and amide and peptide groups (Callis & Liu, 2004 ▶; Permyakov, 1993 ▶; Lakowicz, 2006 ▶)], proton-transfer mechanisms (*i.e.* quenching by tyrosine, lysine and protonated histidine residues; Chen & Barkley, 1998 ▶; Permyakov, 1993 ▶), photo-oxidation mecha­n­isms (*i.e.* quenching by the kynurenine oxidation ring cleavage of tryptophan; Kehoe *et al.*, 2008 ▶) and some further mechanisms that are still under investigation (Permyakov, 1993 ▶).

In this paper, the usefulness of tryptophan as a tool for structural genomics is characterized and the effectiveness of four current prototype UV microscopes to illuminate protein crystals in my laboratory of automated crystallization are reported.

## 2. Materials and methods

### 2.1. Environmental classes of tryptophan

A list of 763 nonhomologous soluble proteins from the PDB was generated. The environmental distribution of 2665 tryptophans from this sample set of crystal structures was analyzed using the programs *PDB*2*ENV* with a probe of radius 0.75 Å (Bowie *et al.*, 1991 ▶) and *DSSP* (Kabsch & Sander, 1983 ▶). When possible, the quaternary structure of the protein was generated using the protein quaternary-structure file server *PQS* (European Bioinformatics Institute, Macromolecular Structure Database; http://pqs.ebi.ac.uk) before analysis.

### 2.2. Genomic analysis

The following genomes were analyzed for tryptophan content: those of the archaea *Aeropyrum pernix* (aero), *Archaeoglobus fulgidus* (aful), *Methanobacterium thermoautotrophicum* (mthe), *Methanococcus jannaschii* (mjan), *Pyrococcus abyssi* (pabyssi) and *P. horikoshii* (pyro) and the bacteria *Aquifex aeolicus* (aquae), *Bacillus subtilis* (bsub), *Borrelia burgdorferi* (bbur), *Campylobacter jejuni* (cjej), *Chlamydia pneumoniae* (cpneu), *C. trachomatis* (ctra), *Deinococcus radiodurans* (dra1), *Escherichia coli* (ecoli), *Haemophilis influenzae* (hinf), *Helicobacter pylori* (hpyl99), *Mycoplasma pneumoniae* (mpneu), *M. genitalium* (mgen), *Mycobacterium tuberculosis* (mtub), *Neisseria meningitidis* (nmen), *Rickettsia prowazekii* (rpxx), *Synechocystis* PC6803 (synecho), *Thermatoga maritima* (tmar), *Treponema pallidum* (tpal) and *Ureaplasma urealyticum* (uure).

### 2.3. Laboratory setup and sampled UV microscopes

My laboratory (http://pepcc.case.edu) contains a Mosquito robot (TTP LabTech, Royston, Herts, England) for drop setting and a RockImager-500 (Formulatrix, Waltham, Massachusetts, USA) for automatic imaging. Four UV microscopes were sampled, one from each of the four leading manufacturers.

Microscope 1: PRS-1000 (Korima, Carsen, California, USA), which consists of two separately mounted cameras, one for brightfield imaging and another for UV imaging, with 5×, 10× and 20× proprietary objectives, a 100 W mercury arc lamp as a light source, a narrow-bandpass filter at 280 ± 10 nm for excitation and a broad-bandpass filter at 350 ± 25 nm for emission.
Microscope 2: UVEX (JANSi, Seattle, Washington, USA), which consists of a single camera unit for brightfield and UV imaging with 5× and 15× quartz with fluorite objectives, LED arrays for light sources, a narrow-bandpass filter at 280 nm for excitation and a broad-bandpass filter at 350 nm for emission.
Microscope 3: MUVIS (Formulatrix, Waltham, Massachusetts, USA), which consists of a single camera unit for both brightfield and UV imaging with a single fixed-zoom 1× silica objective that corresponds to a field of view of 3 mm, LED arrays as light sources, a proprietary bandpass filter for excitation and a broad-bandpass filter at 340–380 nm for emission.
Microscope 4: QDI-2010 Microspectrophotometer (CRAIC Technologies, San Dimas, California, USA), which is a Zeiss retrofitted microscope and consists of two separately mounted cameras, one for brightfield imaging and another for UV imaging, with 10× and 20× (lowest objective ends available) quartz and other pro­prietary material objectives, a 75 W short arc xenon lamp as a light source, a narrow-bandpass filter at 275 ± 10 nm for excitation (epi-fluorescence), a longpass filter at 330 nm for emission (epi-fluorescence) and a bandpass filter at 280 ± 5 nm (absorbance).

### 2.4. Media supplies and their spectral properties

The following brands of media were used in this study: 0.2 mm thick glass cover slips (HR3-231, Lot No. 200801-0679-Ø22*1^Δ^, Hampton Research, Aliso Viejo, California, USA), 0.96 mm thick cover slips (HR3-247 or HR3-515, Hampton Research, California, USA), Mosquito’s ViewDrop II substrate (4150-05600, TTP LabTech), Grace Bio-Labs substrates (45232, Lot No. 090858 and 45233, Lot No. 070841, Grace Bio-Labs, Washington, USA), Formulatrix substrate (prototype), ClearSeal film (HR4-521, Lot No. 8061/MR69150, Hampton Research, California, USA) and CrystalClear HP260 tape (HR4-511, Hampton Research, California, USA). Microscope 4 was used to generate the absorption spectra of the commercial media by placing the media in the light path on the stage, focusing the beam on an area within the range 1–10000 µm^2^.

### 2.5. Purification and crystallization of model proteins

The N-terminal cytoplasmic domain of NBCe1 (NtNBCe1; NP_003750) and full-length inositol 1,4,5-trisphosphate receptor-binding protein (IRBIT; NP_006612), each fused to a noncleavable hexa­histidine tag (MGHHHHHH–) at their N-terminus, were purified using an approach similar to that described in Gill & Boron (2006 ▶). Crystals were grown by the hanging-drop vapor-diffusion method (McPherson *et al.*, 1995 ▶). Crystallization conditions for NtNBCe1 have been described previously (Gill & Boron, 2006 ▶). Crystallization conditions for IRBIT will be described elsewhere. Other crystals in this study were randomly provided by users of the core.

## 3. Results

### 3.1. Bioinformatic analyses of tryptophan residues

**Figure 1:**
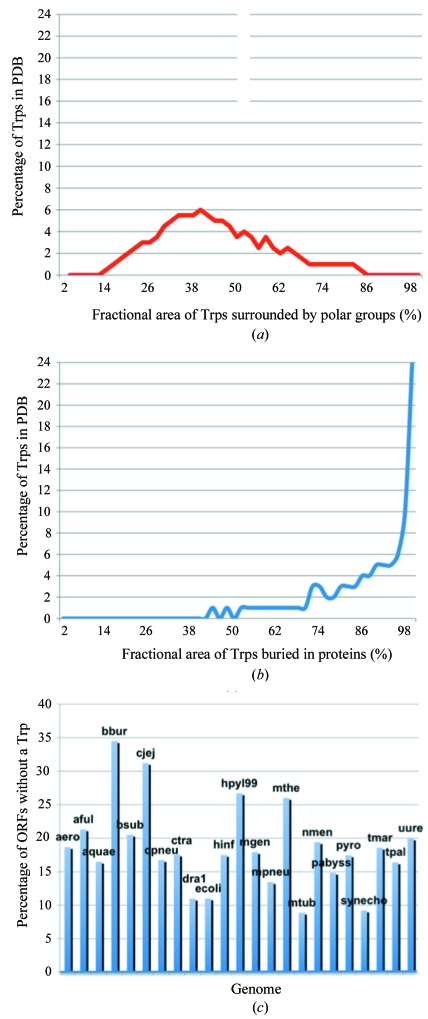
Analyses of tryptophan residues. The environmental distribution of 2665 tryptophans from a sample set of crystal structures show that (*a*) tryptophans tend to be amphipathic in nature, being found in 42.9% ± 16.8% polar environments, and (*b*) have an average of 84.2% ± 19.2% of their surface area buried within proteins. (*c*) The percentage of open reading frames that are without a tryptophan is on average 18.5% ± 6.4% in the shown genomes.

Fig. 1 ▶ characterizes tryptophan residues in known protein structures and in various bacterial genomes. In Fig. 1 ▶(*a*), the ∼43% average surface area of tryptophans that is covered by polar residues is in agreement with data from previous studies by Chothia that characterized the amphipathic nature of tryptophan (Chothia, 1976 ▶). In Fig. 1 ▶(*b*), tryptophans on average have ∼84% of their surface area buried by polar and/or apolar residues in proteins and about 94% of the sampled tryptophans are at least 50% buried. These values are similar to those obtained in a survey by Burley and Petsko, who reported that aromatic pairs are buried or partially buried about 80% of the time (Burley & Petsko, 1985 ▶). Moreover, in genomes from the archaea and bacteria kingdoms, tryptophan residues are calculated here to comprise only 0.5–1.5%, with an average of 1.1% ± 0.3%, of protein residues in any given genome. This range is in agreement with pre-genomic calculations by Wallace and Janes and early post-genomic data values reported by Tekaia and coworkers (Wallace & Janes, 1999 ▶; Tekaia *et al.*, 2002 ▶). In Fig. 1 ▶(*c*), most striking is the high percentage (8.9–34.5%) of open reading frames within any one genome that, according to my tabulation, lack tryptophan.

### 3.2. Certain media attenuate UV light

**Figure 2:**
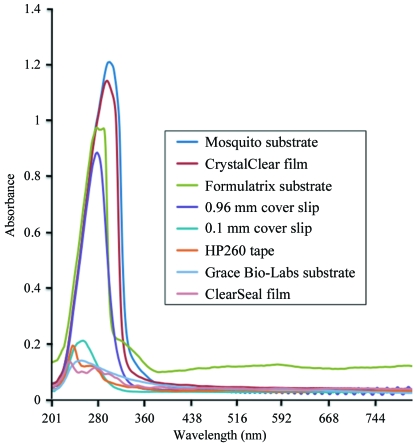
Spectral properties of commonly used media. The spectra of various commercial cover slips, films and substrates for hanging-drop or sitting-drop crystallization experiments are shown. The top-to-bottom order of the legend coincides with the line colors of the graph. We can see that the 0.1 mm cover slip from Hampton Research, the HP260 tape, the substrate from Grace Bio-Labs and the ClearSeal film attenuate UV light minimally and are thus the most effective for high-throughput screening.

Many commonly used covers in crystallography attenuate UV light at 280 nm. Fig. 2 ▶ compares the absorbance spectra of glass, film and substrate covers for hanging-drop or sitting-drop experiments that are widely used in the field. The extent of attenuation varies between the covers. Although UV microscope manufacturers each supply their own list of films for 96-well sitting-drop plates, substrates for 96-­well hanging-drop plates and glass cover slips for VDX plates that work with their microscope, there is little agreement. Some of the inconsistency arises from varying lots of media from third-party manufacturers. Note that for a given medium only particular brands are useful in the UV range.

### 3.3. Extensive exposure of UV light can precipitate protein drops

Unregulated continuous exposure to UV light (in the case of one of the dozen proteins tested here) precipitated the solution containing β-lactamase; that is, over prolonged exposures the protein-containing drop appeared to turn brown, similar to heat-induced denaturation. Of the four microscopes used in this study, microscopes 1 and 4 best illustrated this point. Unlike microscopes 2 and 3, which have computer-controlled exposure times (default UV-exposure settings of 1–2 min), microscopes 1 and 4 do not have an automated shutter for the UV light; the lamps are manually switched. The protein drop is continuously exposed to UV light as the user obtains images. Indeed, the pioneering microscope 1 destroyed a drop during a demonstration after an exposure of ∼5 min.

### 3.4. Differences in fluorescence sensitivity among microscope manufacturers

**Figure 3:**
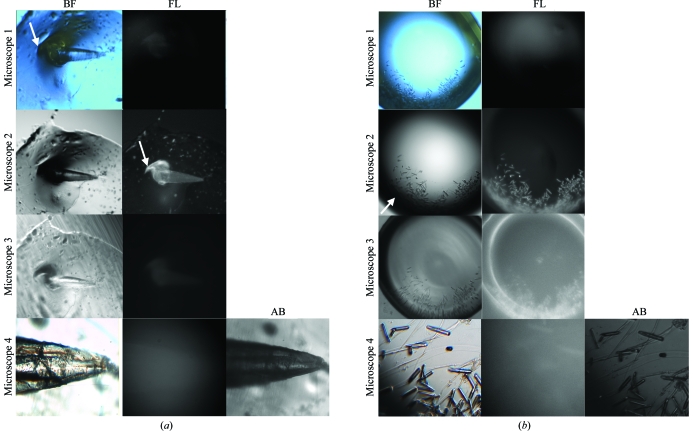
A difficult test to compare the abilities of UV microscopes to detect a protein crystal. Two examples of a protein crystal are shown in hanging-drop experiments. (*a*) A crystal of the cytoplasmic domain of the sodium bicarbonate cotransporter (NtNBCe1) is shown. (*b*) Microcrystals of inositol 1,4,5-trisphosphate receptor-binding protein (IRBIT) are shown. Fluorescence (FL): the partial attenuation of UV light by a 0.96 mm glass cover slip distinguished the microscopes based on the quality of their optical hardware to detect emitted light by epi-illumination with 1 s exposures. The ability of microscope 1 to detect emitted light from the crystal is lower than the others, if the camera is indeed detecting any light. Microscope 2 is the most sensitive as is apparent from the bright and sharp images for both crystal examples. Interestingly, there seemingly appears to be a hint of external or reflected light, *i.e.* light that does not originate strictly from the crystal(s) and/or perhaps an enhanced sensitivity of the camera. The FL images for microscope 3 (zero gain) and microscope 4 are similar in intensity but not in clarity. Brightfield (BF): BF images are useful for identifying individual protein and salt crystals when compared with FL images. However, a few of the BF images shown have anomalies: the images from microscope 1 were actually inverted and reverted; here they have been correctly oriented using third-party software. Also, note that the BF image from microscope 2 is significantly dark or black around the rim, masking crystals (see white arrow) and prohibiting usable side-by-side images of BF and UV. Furthermore, note that both microscopes 1 and 4 do not give a one-to-one positional correspondence or register between the BF and FL images owing to the fact that there are two light sources on the camera that are mounted at different locations. Absorbance (AB): microscope 4 can also detect protein crystals by transilluminating UV light from the bottom of the tray and detecting from the top of the microscope. Note that microscope 4 clearly identifies the NtNBCe1 crystal as protein by AB, thereby suggesting that AB is a more robust method than FL. However, microscope 4 is not able to clearly identify IRBIT as protein by AB, perhaps owing to the location of microcrystals at the edge of the drop, thereby showing some limitation under these conditions (see Fig. 4 ▶).

As shown in Fig. 3 ▶, microscope 2 appears to be the most sensitive as judged by the apparent fluorescence of the crystal. This observation is rather surprising given the significant attenuation of the excitation UV light by a 0.96 mm thick glass cover slip (see Fig. 2 ▶).

All the manufacturers have yet to measure accurately the flux of their microscopes either at the objective or at the sample. A description of the light sources, filters and objectives for each microscope is provided in §[Sec sec2]2. To ascertain whether the apparent fluorescence from microscope 2 arises from a larger flux of UV light compared with the other microscopes, a white fluorescent paper was placed in focus in each UV microscope. The flux from microscope 1 actually appeared to be the most intense and tightly focused: it was more than twice as bright and half as broad compared with microscopes 2, 3 and 4. However, the high flux from microscope 1 does not result in a higher signal-to-noise ratio or increased fluorescence compared with the other microscopes. Longer 3 s exposures with the intense flux of microscope 1 still do not seem to improve the contrast between fluorescence and background, suggesting that the camera system of microscope 1 is inefficient.

The difference in sensitivity among the microscopes also correlated with the differences in the objectives among the microscopes. Unlike the other microscopes, microscope 3 only uses a low-magnification objective, which made it difficult to identify fluorescent crystals. Higher magnification objectives generally have a higher numerical aperture (NA), which improves fluorescence and image quality in epi-illumination. This in agreement with textbook knowledge that both the amount of excitation light delivered to the object and the amount of fluorescent light collected from the object rise with the square of NA, so that the overall advantage rises with the fourth power of NA (Inoué & Spring, 1997 ▶). In addition, the clarity of the images from microscopes 2 and 4 were better than those from microscope 3, whose objective may not be of optimal design.

**Figure 4:**
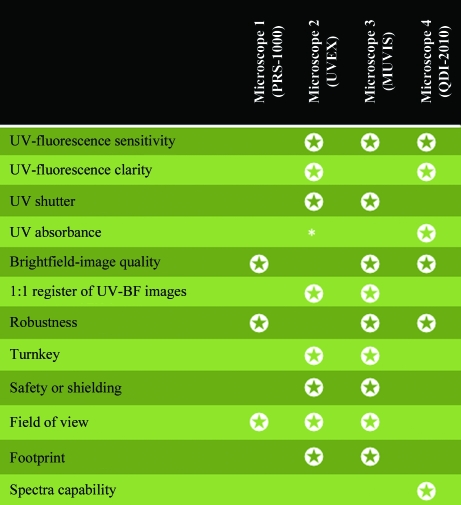
Comparison of prototype UV microscopes. Each model selected demonstrates an important feature when evaluating the efficacy of a UV microscope. The star ratings indicate the featural strengths of a microscope. List prices for each microscope are as follows: microscope 1, $80 000; microscope 2, $55 000–$80 000; microscope 3, $35 000; microscope 4, $200 000. * denotes work in progress. The word turnkey means that the microscope is ready to operate without extensive training. Other device types for UV–Vis monitoring of protein crystals (not part of this study) include the DUVI, which is part of the SpetroImager-501 system that measures dynamic light scattering *in situ* (Dierks *et al.*, 2008 ▶; Meyer *et al.*, 2009 ▶), and the Minstrel HT UV (Rigaku, Carlsbad, California, USA). Other device types for X-ray diffraction screening of protein crystals *in situ* include the recently developed Oxford Diffraction PX scanner (Varian, Palo Alto, California, USA) and the robotic arms that hold the crystallization plate vertical to a synchrotron source (Jacquamet *et al.*, 2004 ▶) as implemented, for example, on the FIP beamline at the European Synchrotron Radiation Facility (Roth *et al.*, 2002 ▶).

Fig. 4 ▶ summarizes other comparisons of each UV microscope, highlighting the featural strengths of each manufacturer.

### 3.5. Crystals and noncrystalline materials alike fluoresce

Despite the above drawbacks of the fluorescent approach, several drops containing crystalline and noncrystalline material (generated in high-throughput screens) fluoresced under UV light. In a metal-additive screen for crystallization of the cytosolic carbonic anhydrase domain of RPTP-γ, the fluorescence of crystalline objects shaped like smooth stones helped to identify the objects as protein. These stone-like objects, which were initially dismissed as salts, now suggest that the domain requires the presence of a metal ion for crystallization. In a screen for the crystallization of the protein thalin, the fluorescence of the instantaneously formed needles identified them as protein and so allows one to initiate, with minimal delay, new crystallization trials for optimization. The UV microscopes also illuminate clusters of protein, such as fibers, spherulites and amorphous material, which all pre­sumably consist of higher concentrations of protein than the background of the drop. Note the bright feather-like material on top of the crystal in Fig. 3 ▶ shown with microscope 1 (see white arrow in brightfield column), microscope 2 (see white arrow in fluorescence column) and microscope 3. We can see from this feather-like material that the UV light does not distinguish between protein crystals and noncrystalline protein objects in the drops.

### 3.6. Tryptophan fluorescence *versus* tryptophan absorbance

**Figure 5:**
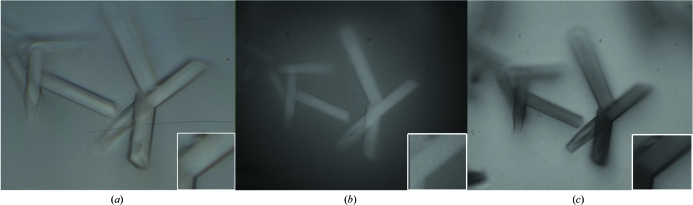
Comparing tryptophan fluorescence with tryptophan absorbance. The cover slip holding the IRBIT protein crystal in Fig. 3 ▶(*b*) is now flipped, removed from the tray and directly exposed to BF (*a*) and UV (*b*, *c*) light microscopy. Here, the crystals are confirmed to be protein (*i.e.* knowing that there is no other aromatic component in the crystallization condition) by tryptophan fluorescence (bright crystals in *b*) and by tryptophan absorbance (black-colored crystals in *c*). If the crystals were salt they would appear dark in (*b*) and white in (*c*).

Fig. 5 ▶ shows a comparison between fluorescence and absorbance on a sample protein crystal. Although none of the microscopes work well on a genomic scale of screening by tryptophan fluorescence, especially with the currently available media for hanging drops, microscope 4 allows us to analyze by either fluorescence (epi-illumination) or absorbance (transmission). Note the following. (i) The fluorescing light in Fig. 5 ▶(*b*) appears to be flat and homogenous throughout the crystals, as one would expect for light only arising from the crystal. (ii) The light emitted from the crystals is diffuse (inset in Fig. 5 ▶ *b*), while the light absorbed from the crystals (inset in Fig. 5 ▶ *c*) is sharp. That is, after excitation the emitted light of fluorescence scatters in all directions with interference by the surrounding protein solution, while the absorbed light travels within the crystals unidirectionally without significant interference by the protein solution. (iii) Tryptophan absorbance is better at detecting the crystals at the corners of the drop than brightfield or tryptophan fluorescence.

## 4. Discussion

### 4.1. When is UV useful?

Although the results above demonstrate that UV indeed appears to be invasive or damaging to protein in some cases, it does rapidly identify crystals in high-throughput screens. UV is also useful for identifying leads to protein crystallization, separating the many amorphous materials that are typically generated by screens into the categories of either protein or artifact. Screens are only meant to be a starting point for crystallization.

That said, there are a growing number of known situations in which UV fluorescence may not identify a protein crystal. Firstly, nearby groups around tryptophan may be able to quench the fluorescence of the indole chromophore (Dose, 1968 ▶; Permyakov, 1993 ▶; Lakowicz, 2006 ▶; Kehoe *et al.*, 2008 ▶). Fig. 1 ▶(*a*) suggests that the fluorescence signal of an average tryptophan might already be subjected to partial quenching by surrounding residues. However, other quenching factors may also contribute to quenching, such as co­factors, metals or other additive molecules in near proximity to tryptophans. Examples of cofactors or additives in proteins that have been observed to quench tryptophan and/or tyrosine fluorescence include heme groups, succinimide, adenine, saccharin, certain detergents and 2-­mercaptoethanol (Permyakov, 1993 ▶; Hof *et al.*, 1996 ▶). Secondly, in genomes from the archaea and bacteria kingdoms, Fig. 1 ▶(*c*) shows that up to approximately one-third of the genomic open reading frames can be deficient in tryptophan. In humans, there are also instances, such as insulin, calmodulin, troponin C or ribonuclease A (mature version), where the protein sequence may not contain tryptophan, or it may be devoid of both tryptophan and tyrosine, such as in most parvalbumins and green-pea superoxide dismutase (Permyakov, 1993 ▶). Thirdly, fluorescence may also arise from anomalies of the crystallization trial in which the protein concentration in the solution is higher than the background, such as the bright feather-like material in Fig. 3 ▶. As a note, this situation of higher areas of protein concentration within a drop is different from the situation of a drop with heavy precipitation in solution that covers or masks microcrystals. The fluorescence of the precipitate should be minimal compared with the fluorescence of microcrystals or areas where the protein concentration is higher than the background because the tryptophans of unfolded protein are directly exposed to solvent and their fluorescence is quickly quenched. Hence, for all the above reasons the minimum fluorescent signal that positively identifies a protein crystal has yet to be quantitated.

### 4.2. Why do some proteins precipitate under UV light?

A strong or continuous amount of UV absorption in proteins could lead to the formation of free radicals by bond breakage, perhaps through increased kinetic energy of atom or bond vibrations, resulting in structural changes or precipitation. For example, tryptophan and tyrosine absorb UV light strongly on excitation at the 280 nm wavelength. As exemplified by Eisenberg and Crothers, for a protein that contains two tryptophans and six tyrosines we can calculate using the Beer–Lambert law that 75% of UV light is absorbed and 25% is transmitted by a 1 mg ml^−1^ protein solution concentration in a cuvette with path length 0.1 mm (Eisenberg & Crothers, 1979 ▶). The strong aborption of UV light by these residues could result in photolysis (Dose, 1968 ▶) or photo-oxidation (Kehoe *et al.*, 2008 ▶) mechanisms that could lead to protein unfolding and subsequent unfavorable cross-linking. Keheo and cowokers reported the aggregation of β-lactoglobulin that resulted from the cleavage of disulfide bridges and photo-oxidation of tryptophan to *N*′-formyl­kynurenine and that resulted in an increase in exposed sulfhydryl groups (Kehoe *et al.*, 2008 ▶). Permyakov described an electron-transfer mechanism in α-lactalbumin between tryptophan and a nearby disulfide that resulted in reduction of the disulfide bonds (Permyakov, 1993 ▶). In another example, despite the absence of tryptophan, Vernede and coworkers demonstrated using X-ray crystallo­graphic methods the damage to the CysA7–CysB7 disulfide bridge in insulin by UV radiation that resulted in a change in local backbone structure (Vernede *et al.*, 2006 ▶).

### 4.3. Do buried tryptophans present a problem for UV?

Fig. 1 ▶(*b*) shows that the majority of tryptophans are nearly to completely buried. However, the fact that tryptophans might be buried is not in itself a limiting factor for generating UV fluorescence. Solution studies show that tryptophan residues that are hydrophobically buried in the core of proteins have spectra that exhibit a minimal Stokes shift (or have blue-shifted emission spectra) com­pared with tryptophans on the surface of the protein or unfolded protein, which emit at a slightly (∼35 nm) longer wavelength (or have red-shifted emission spectra; Permyakov, 1993 ▶; Lakowicz, 2006 ▶). The spectral properties of tryptophans within a protein crystal should behave similarly to the spectra of proteins in solution. Thus, in light of molecular-dynamics simulations that suggest that tryptophan fluorescence is a consequence of electron transfer from the indole ring to a nearby amide (Callis & Liu, 2004 ▶), the intensity of tryptophan fluorescence should be influenced by degree of freedom on say a loop, contact with solvent and proximity to negatively charged residues or other quenching factors as described above. In this sense, it is to the advantage of UV fluorescence microscopy that tryptophans be buried, preferably in hydrophobic environments.

The real obstacle for fluorescence arises at the boundary of the crystal–solvent interface. The solution surrounding the crystal will contain unknown amounts of free protein molecules that will partially reabsorb any emitted light from the crystal, thereby red-shifting or distorting the true spectral characteristics of the crystal. This is referred to as an ‘inner filter effect’ and is discussed below. This effect impedes studies to directly characterize the UV fluorescence of tryptophans inside a protein crystal unless the crystal is transferred to a non-absorbing solvent such as water.

### 4.4. What types of media should be used in UV microscopy?

Ideally, quartz is the medium of choice for UV transmission at the 280 nm wavelength, although cost makes it prohibitive, especially in high-throughput crystallization trials that will quickly be disposed of. However, in order to overcome cost obstacles, many types of media for general applications have been customized for measurements at a broad range of wavelengths. A high-quality glass is used as the Fura excitation dye filter corresponding to the 340–380 nm wavelengths that are commonly used in calcium absorption measurements. Plastic cuvettes that transmit in the range 220–900 nm allow us to calculate DNA concentration at 260 nm, to calculate protein concentrations at 280 nm and to calculate cell density at 600 nm in spectrophotometers. Given these properties, it is not clear why such plastic materials are not more widely used in the protein-crystallography community.

Manufacturers for UV microscopy are only now working on standardizing the refractive index for their crystallographic plastic consumables and the corresponding wavelength for excitation. Exciting with longer wavelengths, such as 295 nm, generally works better with glass and plastics and minimizes tryptophan absorptivity. Longer wavelengths also diminish the signal from tyrosine and phenylalanine and thus are also useful when monitoring local con­formational changes of protein structure by the fluorescence changes of single tryptophan residues. Conversely, the signal from tyrosine and phenylalanine residues, which are usually present in much higher numbers and are usually present in proteins devoid of tryptophans, could significantly contribute to fluorescence at the shorter 280 nm wavelength when screening for crystals.

### 4.5. What types of media are compatible with automation?

The prerequisite for plastic is not only its ability to transmit UV light; the characteristics and structure of the plastic also have to be tested with complementary equipment for automation. Firstly, the plastic needs to be compatible for general brightfield imaging, say in the RockImager-500, whether the media are for sitting-drop or hanging-drop experiments. This means that the birefringence of the plastic itself has to be minimized. Preferably, the birefringence of the plastic should be eliminated as discussed in Echalier *et al.* (2004 ▶), but even polystyrene trays have some birefringence. Secondly, the plastic has to be compatible with the drop setter. Remarkably, the Mosquito robot is a drop setter on the current market that efficiently produces hanging drops with three protein-to-reservoir solution drops per well at a nanolitre volume. To do this, the Mosquito is dependent on a substrate, a hard plastic consumable that glues itself over a 96-well plate after the drops are set onto it. Although a couple of substrates in Fig. 2 ▶ from third-party manufacturers are UV-compliant (dependent on the wavelength), owing to flimsy materials these manufacturers have yet to implement proper use of these plastics with the Mosquito. Plastics in sitting-drop experiments are less of an issue here.

### 4.6. Is absorbance a better method than fluorescence for detecting crystals?

There are a number of factors that make absorbance slightly preferable over fluorescence when screening.

Firstly, the fluorescence of crystals is relatively low compared with their absorbance. This phenomenon is analogous to what is commonly referred to as another type of inner filter effect in protein solutions. Because of the high concentration or optical density of tryptophans, which absorb strongly with excitation at the 280 nm wavelength near the surface of a crystal, UV light does not uniformly penetrate the crystal and excites only a limited amount of tryptophans. In fluorescence, this means that the majority of the light is emitted primarily from the face of the protein crystal towards the incident light, where partial re-absorption may take place by the surrounding solution that will in turn re-emit at longer (red-shifted) wavelengths. In absorbance, all the light that is absorbed in a protein crystal collectively or additively results in increasing contrast to the background of the drop, resulting in a darkened image. As previously noted, the term ‘inner filter effect’ could imply either a lack of penetration of UV light into the protein crystal or a lack of emission of fluorescence from the crystal owing to re-absorption from the surroundings (Lakowicz, 2006 ▶). It is not clear which effect dominates in protein crystals. In either case, the weak fluorescent light emitted in microscopes 3 and 4 as shown in Fig. 3 ▶ is reasonable and it may be that the camera sensitivity of microscope 2 rather exaggerates the amount of light emitted.

Secondly, if the crystal contains a cofactor that absorbs excitation light, such as ADP (Bagshaw, 2001 ▶), the cofactor will add to the overall absorbance intensity of the crystal and at the same time subtract from the light available for general excitation. As a note, the decreased amount of fluorescent emission that results here because of the absorption (pre-emission) is strictly not referred to as quenching because the quantum yield of fluorescence does not change (Engelborghs, 2003 ▶). In any event, a possible drawback is that any aromatic in solution, say as part of an inhibitor that is used in cocrystallization experiments, will also absorb (or fluoresce) and the crystallization components will have to be scrutinized before assuming that an object in a screen is a protein crystal.

Thirdly, an absorbance image can be obtained in a shorter exposure time than a fluorescent image (Lunde *et al.*, 2005 ▶). In fact, transmission of UV light from the bottom of the tray (penetrating the plastic tray and reservoir solution) nevertheless yielded useable absorbance images.

Fourthly, molecules that absorb will not always fluoresce or will have low fluorescence quantum yields, since excited molecules can dissipate their excitation energy through methods other than light emission, such as through heat dissipation to the solvent (*i.e.* vibrational relaxation), quenching mechanisms and/or nonradiative pathways of decay (Lakowicz, 2006 ▶). For example, DNA crystals absorb but do not fluorescence very well. The fluorescence of aromatic groups (bases) in DNA has been shown to be low in solution (Pisarevskii *et al.*, 1966 ▶) because of alternate pathways of decay (Zhang *et al.*, 2009 ▶).

Fifthly, unlike fluorescence, it may be possible to obtain characteristic spectra of a crystal in a protein drop by absorption, since the loss of excitation energy by the absorption of the surrounding protein solution is negligible compared with that absorbed by a protein crystal. In principle, one might even be able to identify the space groups of multiple-crystal forms in a drop based on the absorption spectra of previously well characterized crystals, although this has yet to be investigated.

Finally, one disadvantage in absorbance is the case of microcrystals covered by heavy precipitate. The precipitate also will absorb and, unlike fluorescence, obscure embedded microcrystals to an extent that will be dependent on the contrast ability of the microscope.

### 4.7. Summary

The number of proteins in a genome that do not contain tryptophan can be significant. Neglecting the contribution of phenyl­alanines and tyrosines, this makes tryptophan less than perfect for detection, depending on the genome.

All microscopes that were tested are advertised as high-throughput units (able to scan a crystallization tray). However, because of the attenuation of UV light by the media (*e.g.* films, some cover slips and plastic substrates) commonly used in sitting-drop and hanging-drop experiments, it was necessary to manually remove or flip the media, minimizing its glare or attenuation, at the same time deleteriously exposing the crystal. Therefore, the implementation for screening by UV is not yet at a point where high throughput can be achieved without consideration of the media.

Tryptophan fluorescence is complicated by the fact that the crystal is in a protein-solution background whose boundaries influence the emitted light. In contrast to tryptophan fluorescence, the net signal from tryptophan absorbance seemed to be less affected by protein environment, requires shorter exposure time for imaging and is demonstrated to give slightly sharper and more conclusive images to distinguish protein from salt crystals than tryptophan fluorescence despite some of the drawbacks of the current media.

## 5. Conclusion

In-depth protein crystal characterization by UV fluorescence is limited owing to the requirement for tryptophan residues, the interference of emission by the protein drop and the concern regarding quenching factors. UV absorbance may be preferable and should be given consideration by the manufacturers. Spectral properties are not strictly necessary for high-throughput screening, however, where an absolute answer (yes or no) is only required to determine the nature of a crystal. Still, in screening core user samples an absolute answer could not always be given without a fiddle factor. The reasons here include poor media choices, unclear fluorescent images and crystalline or thereabouts material that illuminated. While plates themselves are an important factor in brightfield imaging, they are not an important factor in epi-illumination nor do they appear to cause a problem in transmission even when reservoir liquid is present. In terms of the tested microscopes, the exposure times for adequate inspection need only be less than a minute, necessitating the need for a control shutter to minimize protein damage. Performance did not correlate with price. An experienced crystallographer at the helm still proves to be crucial. In a nutshell, UV detection is not likely to make the bridge from identifying a protein crystal to one that actually diffracts X-rays and will not always be conclusive in every case with every microscope model. Nevertheless, its usefulness is apparent every time a protein crystal condition is discovered, when excitement is tempered by nonfluorescence or non-absorption of a salt crystal or as a selection tool to justify time to optimize a preliminary condition.
